# Prevalence and relevance of abnormal glucose metabolism in acute coronary syndromes: insights from the PLATelet inhibition and patient Outcomes (PLATO) trial

**DOI:** 10.1007/s11239-019-01938-2

**Published:** 2019-09-11

**Authors:** Axel Åkerblom, Daniel Wojdyla, Philippe Gabriel Steg, Lars Wallentin, Stefan K. James, Andrzej Budaj, Hugo A. Katus, Anders Himmelmann, Kurt Huber, Agneta Siegbahn, Robert F. Storey, Richard C. Becker, the PLATO Investigators

**Affiliations:** 1 Department of Medical Sciences, Cardiology, Uppsala University, Uppsala, Sweden; 2 Uppsala Clinical Research Center UCR, Uppsala University, Uppsala, Sweden; 3 Duke Clinical Research Institute, Duke University Medical Center, Durham, NC USA; 4 INSERM-Unité 1148, Paris, France; 5 Assistance Publique-Hôpitaux de Paris, Département Hospitalo-Universitaire FIRE, Hôpital Bichat, Paris, France; 6 Université Paris-Diderot, Sorbonne-Paris Cité, Paris, France; 7 NHLI Imperial College, ICMS, Royal Brompton Hospital, London, UK; 8 Postgraduate Medical School, Grochowski Hospital, Warsaw, Poland; 9 Medizinishe Klinik, Universitätsklinikum Heidelberg, Heidelberg, Germany; 10 AstraZeneca Research and Development, Mölndal, Sweden; 11 Department of Cardiology and Intensive Care Medicine, Wilhelminen Hospital, Vienna, Austria; 12 Medical School, Sigmund Freud University, Vienna, Austria; 13 Department of Medical Sciences, Clinical Chemistry, Uppsala University, Uppsala, Sweden; 14 Department of Infection, Immunity and Cardiovascular Disease, University of Sheffield, Sheffield, UK; 15 Division of Cardiovascular Health and Disease, Heart, Lung and Vascular Institute, Academic Health Center, Cincinnati, OH USA; 16 Uppsala Clinical Research Center, Dag Hammarskjölds väg 38, 751 85 Uppsala, Sweden

**Keywords:** Diabetes, Pre-diabetes, Hemoglobin A1C, Acute coronary syndromes, Myocardial infarction, Risk prediction

## Abstract

Diabetes mellitus (DM) and abnormal glucose metabolism are associated with cardiovascular (CV) disease. We investigated the prevalence and prognostic importance of dysglycaemia in patients with acute coronary syndromes (ACS) in the PLATelet inhibition and patient Outcomes (PLATO) trial. Diabetes was defined as known diabetes or HbA1c ≥ 6.5% or non-fasting glucose ≥ 11.1 mmol/L on admission, prediabetes as HbA1c ≥ 5.7% but < 6.5%, and no diabetes as HbA1c < 5.7%. The primary endpoint was the composite of CV death, spontaneous myocardial infarction type 1 (sMI) or stroke at 12 months. Multivariable Cox regression models, adjusting for baseline characteristics, and biomarkers NT-proBNP and troponin I, were used to explore the association between glycaemia and outcome. On admission, 16,007 (86.1%) patients had HbA1c and/or glucose levels available and were subdivided into DM 38.5% (6160) (1501 patients had no previous DM diagnosis), prediabetes 38.8% (6210), and no DM 22.7% (3637). Kaplan Meier event rates at 12 months for CV death, sMI or stroke per subgroups were 14.5% (832), 9.0% (522), and 8.5% (293), respectively with multivariable adjusted HRs, versus no diabetes, for diabetes: 1.71 (1.50–1.95) and for prediabetes 1.03 (0.90–1.19). Corresponding event rates for CV death were 6.9% (391), 3.4% (195) and 3.0% (102), respectively, with adjusted HRs for patients with DM of: 1.92 (1.42–2.60) and for prediabetes 1.02 (0.79–1.32). Abnormal glucose metabolism is common in ACS patients, but only patients with definite DM have an increased CV risk, indicating that prediabetes is not immediately associated with worse CV outcomes.

## Highlights

- Abnormal glucose metabolism is common in ACS patients.
- Patients with definite diabetes have a doubled CV risk during the first year after an ACS event.
- However, prediabetes is not immediately associated with worse CV outcomes during up to one year of follow up.
- Although prediabetes is important to diagnose, the CV risk is not increased, compared with normoglycemic patients, during the first year after ACS.

## Introduction

Diabetes mellitus (DM) is associated with a two- to four-fold increased risk for cardiovascular (CV) events compared with non-diabetics [1]. In patients without diagnosed DM, but with borderline increased haemoglobin A1c (HbA1c), heightened fasting glucose or abnormal glucose tolerance test, an increased risk of CV events has been observed in a general population [2, 3]. The impact of dysglycaemia on CV risk in patients with acute coronary syndrome (ACS) is less clear with partly conflicting results [2–10]. In the PLATelet inhibition and patient Outcomes (PLATO) trial, we evaluated the effect of the antiplatelet drug ticagrelor, compared to clopidogrel, in patients with ACS [11, 12]. We have previously reported ticagrelor to be both more efficacious and of similar safety compared with clopidogrel, irrespective of diabetes status [8]. In the current substudy, we stratified patients into clinically accepted subgroups of glycaemia based on admission non-fasting glucose and HbA1c to explore the impact of dysglycaemia on CV outcomes [3, 8].

## Methods

### Study population

The PLATO trial (NCT00391872) enrolled 18624 patients with ACS, who were randomised to either clopidogrel or ticagrelor treatment in addition to optimal medical therapy, including aspirin, and optional invasive strategy [11, 12]. All patients were evaluated for diabetes at baseline, and baseline non-fasting glucose concentration and HbA1c were collected. All participants provided written informed consent and the study complied with the declaration of Helsinki. The study was approved by all local Ethics Committees and Institutional Review Boards.

### Laboratory analysis

The details of laboratory sampling and handling have previously been published [11, 12].

Plasma glucose and HbA1c concentrations were determined with sandwich immunoassays on the Cobas® Analytics e601 Immunoanalyzer (Roche Diagnostics, Mannheim, Germany) at the Uppsala Clinical Research Center laboratory, Uppsala, Sweden.

### Definitions of outcome events and glycaemia subgroups

The primary endpoint of the present biomarker substudy was the composite of CV death, stroke or spontaneous myocardial infarction (sMI) within one year of follow up. sMI was defined in accordance with the universal definition of myocardial infarction (MI), hence a non-procedure-related, non-fatal, MI type 1 [13].

DM was defined as patients with known diabetes or admission HbA1c ≥ 6.5% (≥ 48 mmol/mol), or non-fasting venous glucose ≥ 11.1 mmol/L on admission. Pre-DM was defined as patients with HbA1c ≥ 5.7% but < 6.5% (≥ 39 mmol/mol but < 48 mmol/mol) [3]. Patients with HbA1c < 5.7% were considered to have neither DM nor pre-DM (referred to here as ‘non-diabetes’) [3].

### Statistical analysis

**Fig. 1:**
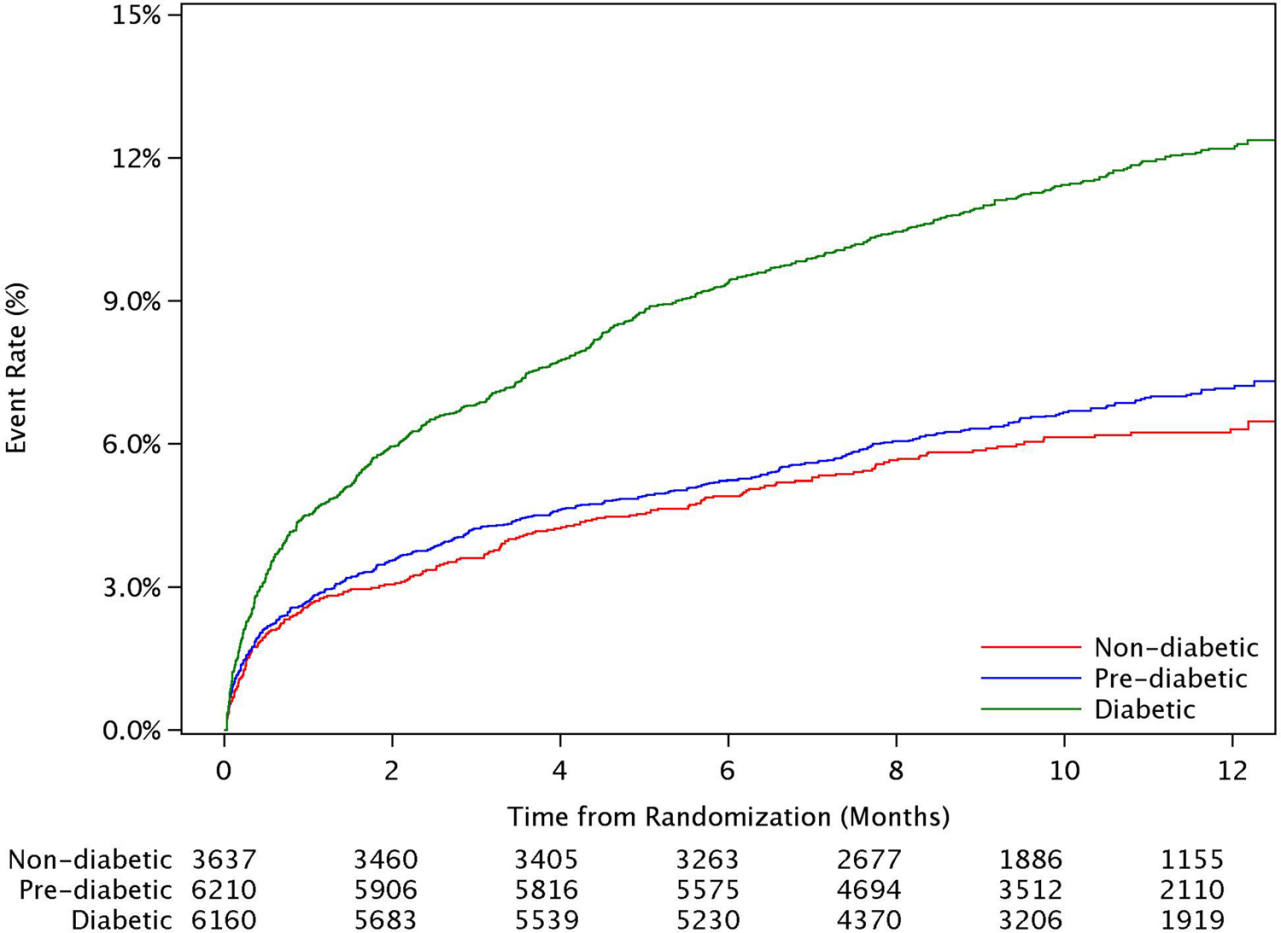
Kaplan Meier plots of the composite endpoint per glycemic subgroups

Baseline characteristics were presented by subgroups of glycaemia, and compared using chi-square or Kruskal–Wallis tests. Kaplan Meier event rates at 1-year, overall and by glycaemic subgroups, were estimated. The associations between glycaemic subgroups, with the non-diabetes group as reference, and the outcomes were assessed by Cox proportional hazard models. Unadjusted and adjusted hazard ratios were derived. Adjustment variables included baseline characteristics (age, gender, smoking status, type of ACS, and history of angina, MI, hypercholesterolemia, diabetes, hypertension, chronic kidney disease, previous; percutaneous coronary intervention (PCI), coronary artery bypass grafting (CABG); stroke or transient ischemic attack (TIA), as well as log transformed biomarkers; NT-pro-BNP and hs-troponin I. p-values less than 0.05 were considered statistically significant results. Statistical analyses were performed with SAS 9.4 (SAS Institute, Cary, NC) (Fig. 1).

## Results

Information on previous DM, non-fasting glucose and/or HbA1c, were available for 16,007 patients (85.9%). On admission, 6160 patients (38.5%) had DM according to our pre-specified criteria, of which 1501 patients (9.4%) had no previous DM diagnosis, consequently new-onset DM. In the new-onset DM subgroup, 1076 patients had an HbA1c ≥ 6.5%. Another 201 individuals had non-fasting blood glucose above 11.1 mmol/L, but with normal HbA1c. A total of 224 patients had both an elevated HbA1c and non-fasting blood glucose above 11.1 mmol/L.

Patients with DM were older, and had more comorbidities compared to patients without DM (Table 1). In patients with DM, normal HbA1c (< 5.7%) was observed in 146 patients (2.4%), while HbA1c was between 5.7 and 6.4% in 811 patients (13.2%) and 4346 patients (70.5%) had an HbA1c of ≥ 6.5%. HbA1c was not available in 857 patients (13.9%) classified as diabetic by other criteria.

**Table 1:** Baseline characteristics by glycemic subgroups

Characteristic	Diabetic (N = 6160)	Pre-diabetic (N = 6210)	Non-diabetic (N = 3637)	Overall (N = 16,007)	p-value
Female gender	2074 (33.7%)	1703 (27.4%)	854 (23.5%)	4631 (28.9%)	< 0.0001
Age, years (median, 25th–75th)	64 (56–72)	62 (54–71)	60 (51–68)	62 (54–71)	< 0.0001
Weight, kg (median, 25th–75th)	81.0 (70.0–92.0)	80.0 (70.0–90.0)	78.0 (68.0–86.0)	80.0 (70.0–90.0)	< 0.0001
Smoking status					< 0.0001
Non-smoker	2794 (45.4%)	2285 (36.8%)	1273 (35.0%)	6352 (39.7%)	
Ex-smoker	1674 (27.2%)	1481 (23.8%)	875 (24.1%)	4030 (25.2%)	
Habitual smoker	1692 (27.5%)	2444 (39.4%)	1489 (40.9%)	5625 (35.1%)	
Systolic blood pressure (mmHg)	135 (120–150)	130 (120–150)	130 (120–149)	133 (120–150)	< 0.0001
Diastolic blood pressure (mmHg)	80 (70–88)	80 (70–90)	80 (70–90)	80 (70–90)	0.0222
*Medical history*					
Angina pectoris	3211 (52.1%)	2734 (44.0%)	1361 (37.4%)	7306 (45.6%)	< 0.0001
Myocardial infarction	1565 (25.4%)	1180 (19.0%)	585 (16.1%)	3330 (20.8%)	< 0.0001
Percutaneous coronary intervention	1049 (17.0%)	738 (11.9%)	385 (10.6%)	2172 (13.6%)	< 0.0001
Coronary artery bypass graft	546 (8.9%)	293 (4.7%)	137 (3.8%)	976 (6.1%)	< 0.0001
Non-haemorrhagic stroke	324 (5.3%)	206 (3.3%)	99 (2.7%)	629 (3.9%)	< 0.0001
Hypertension	4827 (78.4%)	3787 (61.0%)	1977 (54.4%)	10,591 (66.2%)	< 0.0001
Dyslipidaemia	3389 (55.0%)	2706 (43.6%)	1446 (39.8%)	7541 (47.1%)	< 0.0001
Peripheral artery disease	514 (8.3%)	335 (5.4%)	165 (4.5%)	1014 (6.3%)	< 0.0001
Chronic renal disease	416 (6.8%)	180 (2.9%)	99 (2.7%)	695 (4.3%)	< 0.0001
*Treatment approach*					< 0.0001
Invasive	4184 (67.9%)	4489 (72.3%)	2718 (74.7%)	11,391 (71.2%)	
*Type of ACS*					< 0.0001
STEMI	2183 (35.4%)	2628 (42.3%)	1582 (43.5%)	6393 (39.9%)	
*Medications at the time of randomization*					
Aspirin	5800 (94.2%)	5834 (93.9%)	3387 (93.1%)	15021 (93.8%)	0.1056
Beta blockers	4307 (69.9%)	4382 (70.6%)	2545 (70.0%)	11234 (70.2%)	0.7072
ACE inhibitors	3885 (63.1%)	3400 (54.8%)	186 (51.1%)	9145 (57.1%)	< 0.0001
Angiotensin II receptor blockers	764 (12.4%)	445 (7.2%)	247 (6.8%)	1456 (9.1%)	< 0.0001
Statins	4936 (80.1%)	4978 (80.2%)	2865 (78.8%)	12,779 (79.8%)	0.1904
*Biochemistry (median, 25th–75th)*					
Creatinine clearance^a^ (mL/min)	76.9 (58.8–96.5)	80.8 (64.1–98.5)	84.3 (67.2-102.3)	80.4 (63.0–99.0)	< 0.0001
Glucose (mmol/L)	9.3 (7.0–12.5)	6.4 (5.6–7.5)	6.1 (5.4–7.1)	6.9 (5.7–8.8)	< 0.0001
Haemoglobin A1c (%)	7.2 (6.5–8.5)	5.9 (5.8–6.1)	5.5 (5.3–5.6)	6.0 (5.7–6.6)	< 0.0001
NT-proBNP (pmol/L)	76 (24–244)	53 (18–164)	49 (17–147)	59 (19–184)	< 0.0001
High sensitivity troponin I (ng/Lµg/L)	2.0 (0.2–11.8)	2.0 (0.2–12.0)	2.2 (0.2–12.2)	2.1 (0.2–12.0)	0.4910

^a^Cockcroft Gault

In addition, 6210 (38.8%) patients had pre-DM defined as HbA1c concentrations of ≥ 5.7% but < 6.5%. Normal HbA1c concentrations (HbA1c < 5.7%) were observed in 3637 (22.7%) of the study population.

### Glycaemia subgroups and outcome

The Kaplan Meier event rates at 12 months for the combined endpoint of CV death, sMI or stroke per subgroups were 12.2% (694) for patients with DM, 7.2% (409) for pre-DM and 6.3% (213) for patients with no DM (Table 2 and Fig 1). In a sensitivity analysis, all MIs (not only sMI) were evaluated in the composite endpoint (CV death, MI and stroke), with event rates at 12 months per subgroups of 14.5% (832) for patients with DM, 9.0% (522) for pre-DM and 8.5% (293) for patients with no DM.

**Table 2:** Proportions, event rates and hazard ratios (HR) per glycemic subgroups

	Diabetes	Pre-diabetes	No diabetes
Proportion and number of patients	38.5% (6160)	38.8% (6210)	22.7% (3637)
of which were new diagnosed	9.4% (1501)		
Event rates at 12 months			
CV death, sMI or stroke	12.2% (694)	7.2% (409)	6.3% (213)
CV death	6.9% (391)	3.4% (195)	3.0% (102)

	HR (95%CI)	HR (95%CI)	
HR versus no diabetes			
CV death, sMI or stroke	1.97 (1.69–2.29)	1.12 (0.95–1.32)	1
CV death	2.30 (1.85–2.86)	1.12 (0.88–1.42)	1
Adjusted HR vs no diabetes (n = 14,033)			
CV death, sMI or stroke	1.39 (1.11–1.74)	1.02 (0.86–1.22)	1
CV death	1.92 (1.42–2.60)	1.02 (0.79–1.32)	1

The association between glycaemia subgroups and the combination of CV death, sMI or stroke is presented in Table 2.

The Kaplan Meier event rates for CV death alone were: 6.9% (391), 3.4% (195) and 3.0% (102), with adjusted HRs presented in Table 2.

The individual components of the composite endpoint were evaluated in secondary analysis and are presented in Table 2.

## Discussion

DM is independently associated with CV events, but there are conflicting reports on the impact of pre-DM on outcomes [2–7, 9]. This may be due to differences in the identification, classification, follow-up and, in some cases, a lack of other important prognostic variables in ACS, including hs-troponin and NT-proBNP [5]. DM is a multifaceted syndrome, and the pathogenesis for CV disease may include macrovascular changes, endothelial cell dysfunction and accelerated atherosclerosis, with a subsequent increased risk for plaque rupture, thrombus formation and ultimately vessel occlusion [3].

In the current substudy, we found comorbidities to be more common in patients with DM, including disadvantageous biomarker levels such as elevated serum creatinine and NT-proBNP, indicating underlying organ dysfunction. We did not, however, observe any differences between patients with pre-DM and patients without DM.

Pre-DM, defined as blood glucose concentrations higher than normal but not meeting the definition of diabetes, is associated with an approximately 5–10% annual risk of developing type 2 DM [14]. When diagnosed with DM, the patient momentarily is considered to have heightened risk for adverse events, and the indication for cardiac revascularisation, medical treatments e.g. ACE inhibitors, and fluid therapy with angiography uses the presence of DM as a dichotomous condition [2, 3, 15]. It is, however, unknown if the observed heightened CV risk starts even at moderate levels of dysglycaemia or if abnormal glucose metabolism below a certain threshold may be non-pathogenic [3, 14].

In the current cohort, the proportion of patients with DM and at the same time a normal HbA1c was 2.5%, indicating that medical and lifestyle treatments still are not able to maintain normal HbA1c in patients with diabetes.

The increased CV risk for patients with manifest DM was, however, not observed in patients with pre-DM during the first year after ACS, and we believe this highlights that a direct effect of hyperglycaemia on clinical outcome may not be present, rather the deleterious hyperglycaemic effects may arise after a prolonged and accumulated exposure to dysglycemia.

### Limitations

Manifest DM was one of several enrichment criteria employed to enter the PLATO trial if presenting with a non-STEACS [11, 12]. It is likely that the current population exhibited a larger proportion of patients with diagnosed DM than other ACS populations, and consequently proportionally smaller subgroups of patients with pre-DM and no DM. Although follow up was up to one year, the detrimental effect of DM was not observed within the first year of diagnosis in the current study, however this study was not designed to answer these important questions.

## Conclusions

Abnormal glucose metabolism is common in patients with ACS and 9% of the PLATO study patients had undiagnosed diabetes. Although the overall event rate during the first year after ACS is high, we only observed patients with definite DM to have a further increased CV risk, indicating that pre-DM is not immediately associated with worse CV outcomes over the first year after ACS. Nonetheless, this finding does not diminish the need for close follow-up of patients with pre-DM in order to detect DM early, as recommended by international guidelines.
